# Regional variation of care dependency after hip fracture in Germany: A retrospective cohort study using health insurance claims data

**DOI:** 10.1371/journal.pone.0230648

**Published:** 2020-03-23

**Authors:** Claudia Schulz, Gisela Büchele, Raphael Simon Peter, Dietrich Rothenbacher, Patrick Roigk, Kilian Rapp, Katrin Christiane Reber, Hans-Helmut König

**Affiliations:** 1 Department of Health Economics and Health Services Research, University Medical Center Hamburg-Eppendorf, Hamburg, Germany; 2 Institute of Epidemiology and Medical Biometry, Ulm University, Ulm, Germany; 3 Department of Clinical Gerontology, Robert-Bosch-Hospital, Stuttgart, Germany; University Hospital Zurich, SWITZERLAND

## Abstract

**Objective:**

To investigate variation of care dependency after hip fracture across German regions based on the assessment by the German statutory long-term care insurance.

**Data sources/study setting:**

Patient-level statutory health and long-term care insurance claims data from 2009–2011 and official statistical data from Germany.

**Study design:**

We performed a retrospective cohort study. Investigated multinomial outcome categories were increase in care dependency (new onset or a higher care dependency than pre-fracture), no change as reference and death as competing risk in the quarterly period following hip fracture (follow-up 3 months). Regional variation was operationalized with the variance of regional-level random intercepts based on generalized linear mixed models. We adjusted for patient and regional characteristics.

**Principal findings:**

The study included 122,887 hip fracture patients in 95 German postal code regions. Crude outcomes were 30.87% increase in care dependency and 14.35% death. Results indicated modest variation on regional level. Male sex, increasing age, increasing comorbidity, pertrochanteric and subtrochanteric fracture site compared to femoral neck, time from hospital admission to surgery of 3 or more days, as well as increasing inpatient length of stay, non-participation in rehabilitation and regions with lower hospital density were positively associated with an increase in care dependency.

**Conclusions:**

Several characteristics on patient and regional level associated with the outcome were identified. Variation in the increase in care dependency after hip fracture appeared to be attributable primarily to patient characteristics. Variation on regional level was only modest.

## Introduction

Hip fractures are common consequences of falls of older people. There has been great effort to identify heterogeneity in incidence between countries [1, 2], and within countries [3–6]. This heterogeneity may be caused by different quality of data sources and study designs as well as actual differences between or within countries. In a worldwide comparison, Germany has a fairly high incidence of hip fractures, varying between 249/100,000 [2] and 261/100,000 [1]. Likewise, regional variation of hip fracture incidence within Germany has been investigated [7–9].

Hip fractures can lead to numerous negative health outcomes, including functional impairment [10, 11]. This often is accompanied by care dependency, which implies a decreasing quality of life and increasing health care expenditures. In Germany, formal care dependency is recorded in long-term care insurance claims data, which allows for an analysis of care dependency in hip fracture patients based on claims data. Patient characteristics indicating an increase in care dependency like male sex, increasing age and comorbidities are well-known and were for instance investigated in a former study [12].

There is evidence for regional variation of hip fracture incidence [7–9], but not for regional variation of care dependency after hip fracture. Regional variation of care dependency can be caused by differences in the treatment and care of hip fractures, which can be reflected by demographic or health care supply characteristics of the region. For example, different systems of acute and sub-acute geriatric care are delivered in Germany.

On the other hand, in Germany, there is a guideline [13] that clearly determines the clinical treatment of a hip fracture, which is supposed to be provided nationwide on a standardized level of quality. Moreover, in Germany, long-term care recipients compulsorily have to undergo a standardized assessment in accordance with the German Social Security Code (“Sozialgesetzbuch” (SGB)) XI. The assessment of care dependency is based on required assistance in performing activities of daily living due to illness or disability. The classification is conducted by a qualified physician or nurse of the Medical Service of the Statutory Health Insurance (“Medizinischer Dienst der Krankenversicherung”, MDK) which is the advisory and assessment service of the statutory health and long-term care insurances in Germany. The MDK ensures that all insured persons benefit from the health and long-term care services equally by applying objective standards. This implies a standardized, differentiated and objective measurement of the degree of care dependency. Therefore, patients of similar need for care after hip fracture should be assigned to similar levels of care dependency and inconsistencies regarding this assignment between German regions, if any, should exist only to a very limited degree.

As care dependency is associated with vast use of health care resources (e.g., in Germany there were 3.41 million care recipients causing costs of EUR 35.5 billion in 2017 [14]), analysis of regional variation might uncover local health service gaps and inequalities. The objective of our study was to investigate the regional variation of occurring or increasing care dependency as assessed by the long-term care insurance in hip fracture patients based on German health and long-term care insurance claims data.

## Data and methodology

### Data sources and study population

In Germany, health insurance is mandatory and provides comprehensive protection against health care expenses. About 90% of the population are insured by statutory health insurance, while the remaining 10% have opted for private health insurances, due to self-employment or income above a certain threshold. For the statutory health insurance, contributions are income-related (14.6% of income) and independent of health status. It covers most expenses of inpatient and outpatient treatment as well as pharmaceuticals. There are several different health insurance companies of which all inhabitants may choose one. For all companies, the contribution and the coverage of medical treatment and pharmaceuticals is equal. They only slightly differ in their extra premium (on average 0.9% of income), supply of voluntary additional services and possible bonus programs. The largest association of statutory health insurance companies in Germany is the AOK which covers about one-third of the German population. Health insurance claims data of the AOK are administered by the scientific institute of the AOK (“Wissenschaftliches Institut der AOK”, “WIdO”) which provided patient-related health and long-term care insurance claims data for this study. Data were utilized for the period from 01/01/2009 through 03/31/2012. The index period from 04/01/2009 through 12/31/2011 was used to identify patients with hip fractures due to the hospital admission date. The three months before hip fracture were used as baseline to identify pre-fracture care dependency. The three months after hip fracture were used as follow-up window in order to allow for a temporal relation with hip fracture. Hospital-related and regional data for the reference year 2011 were available from the list of German hospitals [15] and the Federal Statistical Office Germany [16].

### Inclusion and exclusion criteria

All patients insured by the AOK statutory health and long-term care insurance, living in Germany, aged 65 years or older with an incident hip fracture in the identification period were included. Hip fractures were identified using the hospital discharge diagnosis S72.0-S72.2 of the International Classification of Diseases, German Modification (ICD-10) [17]. As our research interest was to investigate increase in care dependency, patients with the highest degree of care dependency (n = 3,701) or decrease of care dependency after hip fracture (n = 297), or with missing information on postal code (n = 35) were excluded.

### Dependent variable

In 1995, a long-term care insurance was introduced in the German social insurance system and is compulsory for all citizens [18]. In order to claim long-term care benefits, people must undergo a compulsory assessment, on which the operationalization of care dependency was based. In Germany, all formal care recipients were categorized in one of three care levels by the MDK based on required assistance in performing activities of daily living. The levels were classified depending on daily time needed for care. Care level 1, 2, and 3 implied requiring basic care such as washing, feeding, or dressing for at least 0.75, 2, and 4 hours daily time, respectively [19]. This classification was the same in all German regions. For the claim of a care level, the date of application by the patient, not the date of decision by the MDK is relevant which makes a short follow-up period reasonable.

In the claims data, information on care level was available only on a quarterly period basis. Therefore, we compared the care level of the quarterly period before and after inpatient treatment of the hip fracture. *Increase in care dependency* was defined as a new onset of care need (i.e. new classification in one of the care levels after fracture) or a higher care level in the quarterly period after the fracture compared to the quarterly period before the fracture. The alternative post-fracture outcome state was *no change in care dependency* (i.e. no classification in one of the care levels after fracture or an identical care level pre- and post-fracture).

Additionally, as mortality is a frequent consequence of hip fractures and affects the outcome states, we included *death* as competing risk.

### Definition of regions

Main analysis was based on the definition of regions by the first two digits of the postal code of patients’ residence. Thus, there were 95 regions with on average 1,294 observed patients per region (minimum 152; maximum 3,352 patients). Additionally, we defined regions based on federal states (16 regions; on average 7,680 patients; minimum 1,157; maximum 19,768) to investigate differences due to administrative borders.

### Independent variables

On regional level, coverage and performance of health care may depend on infrastructure, which might be reflected by population characteristics. Highly populated regions need high-volume hospitals, whereas residents in rural regions have to deal with longer travel distances to the nearest hospital. *Population density* (inhabitants per km^2^/100) served as a proxy variable for the population pattern and the available infrastructure, including for example access to education, transportation, and care.

Furthermore, regional coverage by, as well as competition of, hospitals may affect quality of care. We added *hospital density* (number of hospitals with a surgical department per 100,000 inhabitants) to quantify the number of hospitals available for hip fracture treatment per region.

The application for a care level might, inter alia, be driven by patients’ own life situation and available care supply in the neighborhood. As the reimbursement of expenditures for nursing home care is dependent on the granted care level by the long-term care insurance, the availability and utilization of nursing home care might affect the probability to apply for a care level. Therefore, we adjusted for *nursing home bed density* (number of nursing home beds per 1,000 inhabitants).

Furthermore, rehabilitation may affect functional recovery and care dependency after hip fracture. In Germany, geriatric care including rehabilitative approaches is either delivered as early complex geriatric rehabilitation therapy during index hospitalization on an acute ward (§108/109 SGB V) or as inpatient geriatric rehabilitation in a separate sub-acute rehabilitation facility (§111 SGB V). There are federal states with predominately one of the two geriatric rehabilitation systems and other federal states offering both types. Therefore, we classified the *type of geriatric rehabilitation* offered per federal state in three categories: mainly early complex geriatric rehabilitation; mainly inpatient rehabilitation; or a combination of both forms. The classification was based on the proportion of geriatric beds in acute hospital departments or in sub-acute geriatric rehabilitation facilities for each federal state.

The former two variables *population density* and *hospital density* were available for postal code regions and federal states. The latter two variables *nursing home bed density* and *type of geriatric rehabilitation* were only available on federal state level. All regional-level variables were checked for correlations with each other. Correlations were low and never exceeded 0.31.

On patient level, we took into account *sex*, *age* (as continuous variable), *comorbidities* based on *Elixhauser* [20, 21] and *medication* [22, 23], *time from hospital admission to surgery* (categorized as “0 days”, “1 day”, “2 days”, “3 or more days” and “not applicable” (i.e. no hip fracture surgery was claimed)), *fracture site* (“femoral neck” (ICD-10 diagnosis S72.0), “pertrochanteric” (ICD-10 diagnosis S72.1), “subtrochanteric” (ICD-10 diagnosis S72.2)), *pre-fracture care dependency* (“no care level”, “care level 1”, “care level 2”, “care level 3”) in the quarterly period before hip fracture, *hospital volume* (mean number of hip fracture cases per year in our dataset, weighted with the market share of the AOK per region to avoid bias), participation in *inpatient rehabilitation* within 4 weeks after hospital stay and *inpatient length of stay*. The last variable summed up inpatient days after hip fracture in acute and, if applicable, in sub-acute facilities to account for the two different geriatric treatment systems in Germany taking place either in acute or sub-acute facilities.

### Statistical analysis

For descriptive analysis, rates for increase in care dependency, no change in care dependency and death were calculated as crude share of the total population in the data set and standardized using the sex and age distribution of the diagnosis-specific German hospital population of 2011 with hospital discharge diagnosis S72.0-S72.2 [24].

We used a multinomial logit regression model to estimate the likelihood (odds ratios (OR) with 95% confidence intervals) of the increase in care dependency, compared to no change and death. After the occurrence of increase in care dependency or death, data were censored, which means that if patients died after an increase in care dependency, death was not considered. In order to account for correlation of patients within the same postal code region and to incorporate covariates on both levels, we extended the model through a random intercept per region in order to control for unobserved heterogeneity. We thereby assumed a probabilistic effect of each region on patients, which enabled us to make inferences about variation among all regions [25]. We used a correlated random effects formulation [26–28] to ensure unbiased estimates. We started with an empty model without independent variables, but a random intercept per region. The variance of the random intercepts was supposed to indicate regional-level effects. Additionally, we mutually adjusted for all patient and regional characteristics to avoid overestimation and investigated the effect of regional variables. As not all regional-level information was available for postal code regions, we repeated the analyses considering federal states.

Based on both the unadjusted and adjusted model results, we calculated the predicted probabilities for the outcomes for an average patient with reference (for categorical variables) or mean (for continuous variables) characteristics. We then calculated the predicted probability within 1 and 2 standard deviations (SD) of the regions’ random intercepts, respectively, which enabled us to assess regional-level variation. Due to the random intercepts’ assumed normal distribution with mean 0 and estimated variance, one can find approximately 68% of all observations within 1 SD and 95% within 2 SD from the mean.

The study was approved by the ethics committee of the Ulm University (application number 178/15). Informed consent from the individuals was not needed, as we used anonymized health and long-term care insurance claims data.

## Results

### Descriptive results

The dataset contained 122,887 patients with hip fractures treated in 1,522 hospitals in 95 postal code regions in 16 federal states in Germany. **Tables 1 and 2** show descriptive characteristics of patients in total and stratified by outcome. In total, 30.87% had an increase in care dependency and 14.35% died. Regional-level variables were approximately equally distributed over the outcome categories. About one-quarter of all patients was male and three quarters were female. Mean age was 82.62 years. More than half of patients had no pre-fracture care dependency. Mean Elixhauser comorbidity score was highest for patients who died, and mean medication-based comorbidity score was highest for those with no change in care dependency. Most patients had a femoral neck (48.22%) or a pertrochanteric (44.50%) hip fracture, and had surgery on the same (34.00%) or the next (36.86%) day. Mean inpatient length of stay was longest for patients with no change in care dependency. Overall, 56% of patients received inpatient rehabilitation.

**Table 1:** Baseline characteristics of the study population (N = 122,887).

**Regional-level factors**		
Population density (inhabitants per km^2^/100): mean (SD)	4.10	(6.79)
Hospital density (hospitals per 100,000 inhabitants): mean (SD)	3.33	(10.90)
Nursing home bed density (beds per 1,000 inhabitants): mean (SD)	10.19	(1.55)
Type of geriatric rehabilitation: n (%)		
… mainly early complex geriatric rehabilitation	54,122	(44.03)
… mainly inpatient rehabilitation	50,371	(40.98)
… both forms	18,429	(14.99)
**Patient-level factors**		
Sex: n (%)		
… male	27,980	(22.77)
… female	94,907	(77.23)
Age: mean (SD)	82.62	(7.36)
Pre-fracture care dependency: n (%)		
. . . no care level	64,556	(52.53)
. . . care level 1	35,191	(28.64)
. . . care level 2	23,140	(18.83)
Comorbidity score		
… based on Elixhauser: mean (SD)	2.59	(1.85)
… based on medication: mean (SD)	3.87	(1.99)
Fracture site: n (%)		
… femoral neck, ICD-10: S72.0)	59,259	(48.22)
… pertrochanteric (ICD-10: S72.1)	54,680	(44.50)
… subtrochanteric (ICD-10: S72.2)	8,948	(7.28)
Time from hospital admission to surgery: n (%)		
… 0 days	41,776	(34.00)
… 1 day	45,297	(36.86)
… 2 days	11,721	(9.54)
… 3 or more days	13,873	(11.29)
… not applicable	10,220	(8.32)
Inpatient length of stay: mean (SD)	29.02	(17.12)
Inpatient rehabilitation: n (%)		
… yes	68,839	(56.00)
… no	54,083	(44.00)

SD = Standard deviation. Inpatient length of stay summed up inpatient days in both hospital and rehabilitation facility, if applicable.

**Table 2:** Baseline characteristics stratified by increase in care dependency, no change and death.

N (%)	Increase in care dependency 37,934 (30.87%)		No change 67,321 (54.78%)		Death 17,632 (14.35%)	
**Regional-level factors**						
Population density (inhabitants per km^2^/100): mean (SD)	4.00	(6.61)	4.16	(6.90)	4.06	(6.73)
Hospital density (hospitals per 100,000 inhabitants): mean (SD)	3.17	(10.44)	3.38	(11.06)	3.46	(11.26)
Nursing home bed density (beds per 1,000 inhabitants): mean (SD)	10.18	(1.54)	10.19	(1.56)	10.22	(1.54)
Type of geriatric rehabilitation: n (%)						
… mainly early complex geriatric rehabilitation	16,894	(44.52)	29,242	(43.41)	7,986	(45.28)
… mainly inpatient rehabilitation	15,598	(41.11)	27,772	(41.25)	7,001	(39.71)
… both forms	5,452	(14.37)	10,327	(15.34)	2,650	(15.02)
**Patient-level factors**						
Sex: n (%)						
… male	7,890	(20.80)	14,666	(21.79)	5,424	(30.76)
… female	30,044	(79.20)	52,655	(78.21)	12,208	(69.24)
Age: mean (SD)	84.25	(6.68)	80.96	(7.34)	85.45	(7.16)
Pre-fracture care dependency: n (%)						
. . . no care level	22,533	(59.40)	37,120	(55.14)	4,903	(27.81)
. . . care level 1	12,127	(31.97)	16,517	(24.53)	6,547	(37.13)
. . . care level 2	3,274	(8.63)	13,684	(20.33)	6,182	(35.06)
Comorbidity score						
… based on Elixhauser: mean (SD)	2.37	(1.74)	2.67	(1.85)	3.26	(2.06)
… based on medication: mean (SD)	3.79	(2.00)	4.02	(1.98)	3.84	(1.97)
Fracture site: n (%)						
… femoral neck, ICD-10: S72.0)	16,957	(44.70)	33,694	(50.05)	8,608	(48.82)
… pertrochanteric (ICD-10: S72.1)	17,981	(47.40)	28,950	(43.00)	7,749	(43.95)
… subtrochanteric (ICD-10: S72.2)	2,996	(7.90)	4,677	(6.95)	1,275	(7.23)
Time from hospital admission to surgery: n (%)						
. . . 0 days	13,209	(34.82)	22,966	(34.11)	5,601	(31.77)
… 1 day	13,813	(36.41)	25,269	(37.54)	6,215	(35.25)
… 2 days	3,531	(9.31)	6,452	(9.58)	1,738	(9.86)
… 3 or more days	4,579	(12.07)	7,264	(10.79)	2,030	(11.51)
… not applicable	2,802	(7.39)	5,370	(7.98)	2,048	(11.62)
Inpatient length of stay: mean (SD)	30.64	(16.55)	30.98	(17.20)	18.65	(15.36)
Inpatient rehabilitation: n (%)						
… yes	22,041	(58.10)	43,487	(64.60)	3,292	(18.67)
… no	15,893	(41.90)	23,834	(35.40)	14,340	(81.33)

SD = Standard deviation. Inpatient length of stay summed up inpatient days in both hospital and rehabilitation facility, if applicable.

**Fig 1:**
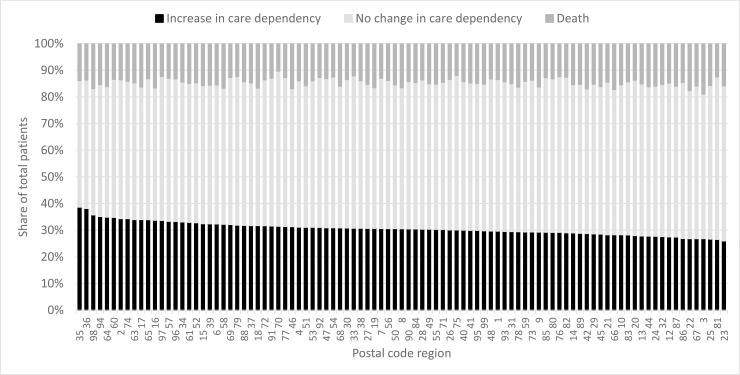
Rates of increase in care dependency, no change and death per 2-digit postal code region. Rates were calculated as share of total patients per region, standardized according to sex and age.

**Fig 2:**
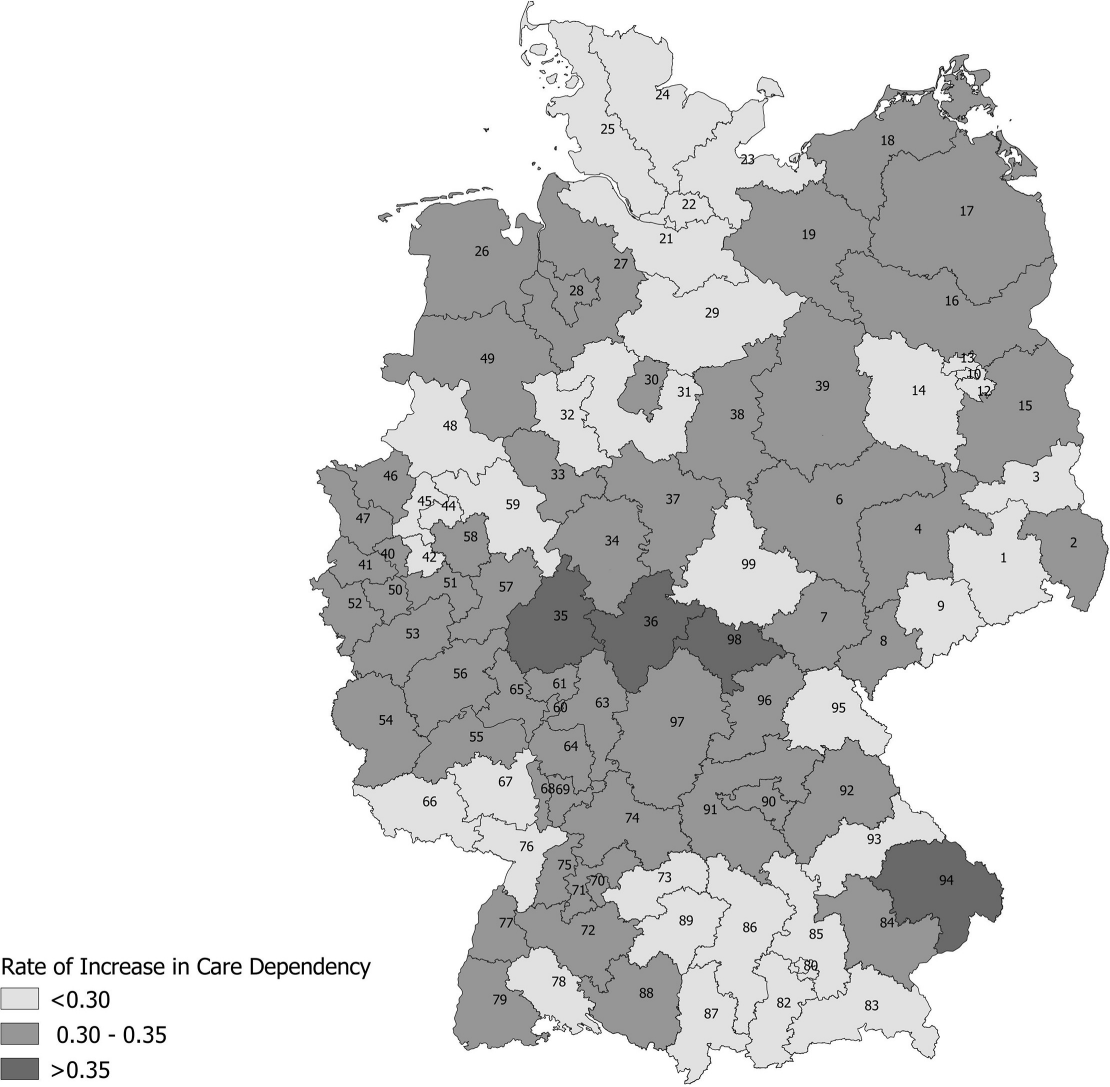
Rates of increase in care dependency per 2-digit postal code region. Rates were calculated as share of total patients per region, standardized according to sex and age.

**Fig 3:**
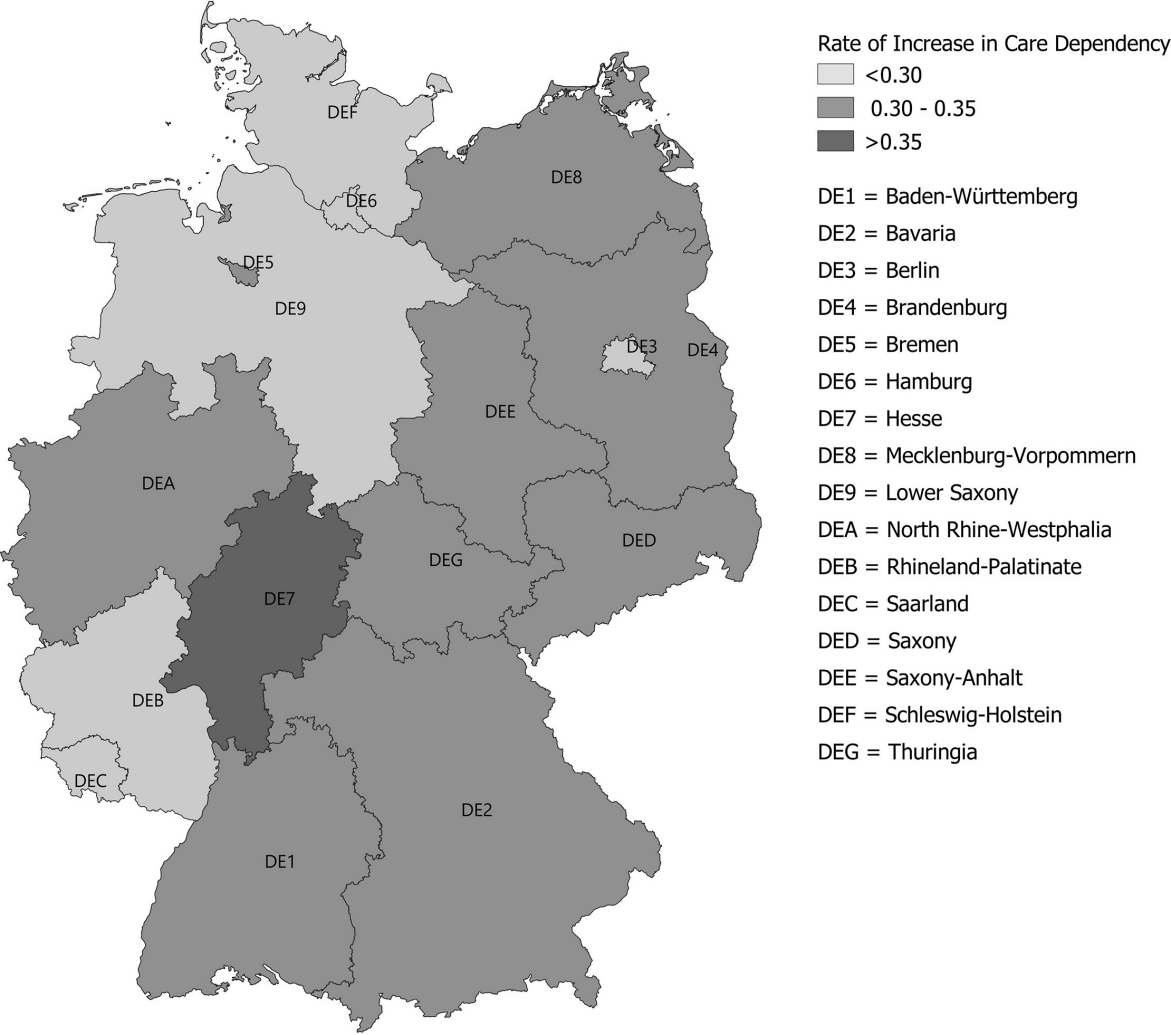
Rates of increase in care dependency per federal state. Rates were calculated as share of total patients per region, standardized according to sex and age.

For each postal code region, age- and sex-standardized rates for the increase in care dependency, no change and death as share of total patients per region are displayed in **Fig 1**. The majority of regions seemed to have roughly similar rates of increasing care dependency, which varied with a range of 5% around the mean of 30.87%. However, there were few outlier regions with considerably higher rates up to 38.72%. Additionally, standardized rates for the increase in care dependency per postal code region were displayed on a map (**Fig 2**). Rates were highest in few regions in middle and south-east Germany and lowest in several regions across the country. Displayed per federal state (Fig 3), rates were highest in Hesse, which stands in line with postal code areas, and lowest in several, but not all, states in the northern and western part of Germany. However, we could not detect any clear regional pattern.

### Multivariate results

Both in the unadjusted and adjusted models, the random intercepts of the postal code regions were significant, which suggested relevant associations due to regional clusters (**Table 3**). However, variance seemed to be rather low (unadjusted: 0.010; adjusted: 0.004). We calculated the predicted probability of an average patient and considered variation within 1 and 2 SD of the random intercepts. As we kept patient-level characteristics constant, all variation would then refer to the 95 postal code regions. Due to assumption of a normal distribution, range within 1 SD included about 68%, and within 2 SD included about 95% of all regions, for which the range of predicted probability for increase in care dependency was 4.85% and 9.71%, respectively. Adjusting for patient characteristic narrowed the range to 2.24% and 4.48%. In other words, patients with otherwise equal characteristics from 68% (95%) of all German postal code regions would have a maximum probability difference of 2.24% (4.48%) to sustain an increase in care dependency after hip fracture. Unadjusted and adjusted variation for death was rather low.

**Table 3:** Variance of regions’ random intercepts and predicted probabilities.

	Regions’ random intercepts		Predicted probability per outcome					Range	
Outcome	Variance	P-value	… - 2 SD	… - 1 SD	… crude	… + 1 SD	… + 2 SD	± 1 SD	± 2 SD	
Increase in care dependency, unadjusted	0.010	< .0001	26.33%	28.51%	30.84%	33.35%	36.04%	4.85%	9.71%	
Death, unadjusted	0.010	< .0001	12.20%	13.20%	14.28%	15.43%	16.67%	2.23%	4.47%	
No change in care dependency (*Ref*)										
Increase in care dependency, adjusted^a^	0.004	< .001	45.36%	46.50%	47.63%	48.74%	49.84%	2.24%	4.48%	
Death, adjusted^a^	0.005	< .01	13.13%	13.52%	13.91%	14.28%	14.65%	0.76%	1.52%	
No change in care dependency (*Ref*)										

Predicted probabilities were calculated for patients with average characteristics and varied within 1 and 2 standard deviations of the postal code regions’ random intercepts.

^a^Adjusted for patient and regional characteristics. Abbreviations: Ref = Reference category, SD = Standard deviation.

In the adjusted model in **Table 4**, *hospital density* (OR = 0.997 per hospital with a surgical department per 100,000 inhabitants in a region) showed a significant negative correlation with increase in care dependency, but *population density* (OR = 0.997 per 100 inhabitants per km^2^ in a region) did not. Further regional-level variables *nursing home bed density* and *share of inpatient geriatric rehabilitation* were only available on a federal state level but did not show significant results in the respective model with random intercepts per federal state (**Table 5**). In **Table 4**, patient characteristics significantly associated with an increased likelihood of increase in care dependency were male *sex* (OR = 1.238), increasing *age* (OR = 1.092 per year from 65), increasing *Elixhauser* (OR = 1.092 per score point) and *medication-based* (OR = 1.092 per score point) *comorbidity scores*, pertrochanteric (OR = 1.175) and subtrochanteric (OR = 1.235) fracture site when compared to femoral neck fracture site, a waiting time from hospital admission to hip fracture surgery of 3 or more days (OR = 1.094), and increasing *inpatient length of stay* (OR = 1.011 per day). *Inpatient rehabilitation* (OR = 0.432) and a not applicable waiting time from hospital admission to hip fracture surgery (OR = 0.847, probably due to death) were associated with a decreased likelihood of increase in care dependency.

**Table 4:** Results of the multinomial logistic regression analysis with random intercepts per 2-digit postal code region.

	Increase in care dependency			Death		
Variable	OR	P-value	95% CI	OR	P-value	95% CI
Intercept	0.056	0.001	0.011–0.288	0.027	0.001	0.003–0.224
**Regional-level factors**						
Population density (inhabitants per km^2^/100; per point increase)	0.997	0.074	0.994–1.000	0.996	0.059	0.992–1.000
Hospital density (number of hospitals per 100,000 inhabitants; per point increase)	0.997	0.009	0.995–0.999	0.997	0.037	0.994–1.000
**Patient-level factors**						
Sex: male *(Ref*: *female)*	1.238	< .0001	1.197–1.280	2.103	< .0001	2.014–2.195
Age (from 65 years on, per year)	1.092	< .0001	1.090–1.095	1.086	< .0001	1.083–1.089
Comorbidity score						
… based on Elixhauser (per score point)	1.092	< .0001	1.084–1.101	1.368	< .0001	1.354–1.382
… based on medication (per score point)	1.092	< .0001	1.084–1.100	0.903	< .0001	0.894–0.912
Fracture site (*Ref*: *femoral neck*, *ICD-10*: *S72*.*0*)						
… pertrochanteric (ICD-10: S72.1)	1.175	< .0001	1.142–1.209	0.994	0.784	0.956–1.035
… subtrochanteric (ICD-10: S72.2)	1.235	< .0001	1.171–1.303	1.070	0.076	0.993–1.154
Time from hospital admission to surgery (*Ref*: *0 days*)						
… 1 day	1.000	0.991	0.969–1.033	1.016	0.506	0.970–1.063
… 2 days	1.010	0.692	0.961–1.062	1.114	0.002	1.040–1.194
… 3 or more days	1.094	< .001	1.044–1.147	1.266	< .0001	1.185–1.353
… not applicable	0.847	< .0001	0.802–0.894	1.111	0.002	1.040–1.187
Inpatient length of stay (per day)	1.011	< .0001	1.010–1.012	0.982	< .0001	0.980–0.984
Inpatient rehabilitation: yes *(Ref*: *no)*	0.432	< .0001	0.416–0.449	0.181	< .0001	0.170–0.192

The model estimated the effect of regional and patient characteristics on increase in care dependency and death (reference category is no change in care dependency), adjusted for pre-fracture care dependency and hospital volume. Abbreviations: OR = Odds ratio estimates, CI = Confidence interval, Ref = Reference category

**Table 5:** Results of the multinomial logistic regression analysis with random intercepts per federal state.

	Increase in care dependency			Death		
Variable	OR	P-value	95% CI	OR	P-value	95% CI
Intercept	0.182	< .0001	0.078–0.423	0.094	< .0001	0.033–0.267
**Regional-level factors**						
Population density (inhabitants per km^2^/100; per point increase)	0.995	0.185	0.988–1.002	1.003	0.473	0.994–1.012
Hospital density (number of hospitals per 100,000 inhabitants; per point increase)	0.929	0.747	0.593–1.455	1.179	0.532	0.703–1.976
Nursing home bed density (nursing home beds per 1,000 inhabitants; per point increase)	0.964	0.166	0.915–1.015	0.967	0.286	0.909–1.029
Type of geriatric rehabilitation (*Ref*: mainly early complex geriatric rehabilitation)						
… mainly inpatient rehabilitation	0.890	0.342	0.701–1.131	0.853	0.262	0.647–1.126
… both forms	0.903	0.213	0.769–1.061	0.928	0.465	0.761–1.133
**Patient-level factors**						
Sex: male *(Ref*: *female)*	1.240	< .0001	1.199–1.282	2.104	< .0001	2.015–2.196
Age (from 65 years on, per year)	1.091	< .0001	1.089–1.094	1.086	< .0001	1.083–1.089
Comorbidity score						
… based on Elixhauser (per score point)	1.092	< .0001	1.084–1.101	1.368	< .0001	1.354–1.382
… based on medication (per score point)	1.092	< .0001	1.084–1.100	0.903	< .0001	0.894–0.912
Fracture site (*Ref*: *femoral neck*, *ICD-10*: *S72*.*0*)						
… pertrochanteric (ICD-10: S72.1)	1.168	< .0001	1.135–1.202	0.995	0.809	0.957–1.035
… subtrochanteric (ICD-10: S72.2)	1.234	< .0001	1.170–1.302	1.065	0.099	0.988–1.148
Time from hospital admission to surgery (*Ref*: *0 days*)						
… 1 day	1.000	0.992	0.969–1.033	1.018	0.428	0.973–1.066
… 2 days	1.008	0.761	0.959–1.059	1.122	0.001	1.047–1.201
… 3 or more days	1.088	< .001	1.038–1.141	1.279	< .0001	1.197–1.366
… not applicable	0.848	< .0001	0.803–0.895	1.121	0.001	1.050–1.198
Inpatient length of stay (per day)	1.011	< .0001	1.010–1.012	0.982	< .0001	0.980–0.984
Inpatient rehabilitation: yes *(Ref*: *no)*	0.436	< .0001	0.420–0.453	0.185	< .0001	0.174–0.197

The model estimated the effect of regional and patient characteristics on increase in care dependency and death (reference category is no change in care dependency), adjusted for pre-fracture care dependency and hospital volume. Abbreviations: OR = Odds ratio estimates, CI = Confidence interval, Ref = Reference category

## Discussion

This study investigated regional variation of new onset or an increase in care dependency after hip fracture as assessed by the long-term care insurance in Germany. The analysis indicated modest variation on regional and rather great variation on patient level for increasing care dependency and death. We found a negative correlation with hospital density, but not with further regional variables. The patient characteristics male sex, increasing age, and increasing comorbidity were strong predictors for an increase in care dependency. Compared to a femoral neck fracture, pertrochanteric and subtrochanteric fractures were associated with an increase in care dependency, as well as a waiting time to the hip fracture surgery of 3 or more days. Inpatient length of stay was positively correlated with an increase of care dependency, and inpatient rehabilitation negatively. However, because of the study design, it remains unclear whether these are causal effects. For example, it is likely that only patients with a potential for functional recovery were selected for rehabilitation and were therefore less care dependent after fracture. Patient-level characteristics were investigated in a former study in more detail [12].

To our knowledge, there is no literature on regional variation regarding care dependency after hip fracture, but regarding variables which may serve as a proxy, e.g. mobility and self-care after hip fracture between municipalities in Norway [29], or changes in functional status after hip fracture between US regions grouped by quintiles of end-of-life expenditures [30]. Low or no variation was detected, which stands in line with our results.

Another study did find differences in self-care and mobility after hip fracture on facility and community level in the US [31]. However, both levels were collapsed into one and it remains unclear to which level differences were attributable. A further study in the US investigated functional status after hip fracture [32] and found regional variation primarily at facility rather than state level. The cluster-level variance may rise with increasing number of clusters, as there are considerable more facilities than states. This may also explain why we found partly significant results of regional variables within 95 postal code regions, rather than within 16 federal states. Hox et al. [33] recommended the smallest acceptable number of clusters to be 30, which we outperformed by using postal code regions, but not by using federal states.

However, each investigated country has a different health care system and the exact process of treatment and aftercare of hip fractures may vary. Therefore, findings from studies from different countries should not be compared without considering this. For example, for the four mentioned studies on care dependency, the outcome was measured using item scores from different instruments (e.g., EQ-5D 3L [29] or FIM motor scores [31, 32]) which were always derived by patient surveys. In our study, however, the degree of care dependency was derived from long-term care insurance claims data in which the patients’ care level is routinely recorded.

The highest degree of care dependency as a consequence of a hip fracture is institutionalization, for which evidence regarding regional variation is available, but differs regarding the approach. One study found that patients injured after a fall would return home after hospitalization less often when living in less deprived, predominantly white or rural areas in England [34]. Another study found that state-level spending for home- and community-based services for delivering long-term care in the US was associated with a decreased risk of nursing home residence for hip fracture patients [35]. A third study found a lower rate of long-term care admissions for hip fracture patients treated in regions with high inpatient rehabilitation rates in Canada [36]. However, those studies vary in their data and methods, and, again, the structure and supply of health care is different for every country, making them difficult to compare. Findings from one country may not be extrapolated to another country without limitations. For example, the precise post-acute pathways and the short- and long-term discharge destination after hip fracture may differ which, of course, may affect a country’s institutionalization rate. In Germany, however, studies on regional variation of nursing home admission after hip fracture are not available at present.

Increased hospital density, a regional-level variable, was associated with a decreased likelihood of occurring or increasing care dependency. Hospital density may serve as a proxy for the degree of care supply per region. The supply is important for the treatment of suddenly occurring injuries which result from external forces, such as falls, and are supposed to be tended immediately. Our results suggest that patients in regions with more hospitals per capita may experience differences in care, probably based on different distances to hospitals or further inpatient health care, which may relate to a decreased likelihood of subsequent care dependency. However, further analyses based on distance measurements should be conducted to investigate this association.

Population density may suggest the regions’ infrastructure and access to health care, which can comprise rehabilitation, follow-up care and even access to prevention. We can assume that hospital and population density will be roughly correlated, although the correlation in our analyses was not critical. As interpretation may in some ways apply for both variables, but as population density lacked significance, it seemed that effects may be primarily attributable to hospitals rather than to general infrastructure.

Although there are two different geriatric rehabilitation systems offered in different German federal states, it was not found to contribute to regional variation. During early complex geriatric rehabilitation therapy, patients receive rehabilitation within few days after surgery to foster early mobilization. Regarding inpatient geriatric rehabilitation, patients stay in hospital for acute treatment and are discharged to afterward receive additional rehabilitation in a separate inpatient unit. The question arises why the concrete implementation of rehabilitation in Germany is not standardized until now. There should be clarification which system, or whether a combination of both, provide best outcomes. A study focusing on a comparison on both systems found that inpatient geriatric rehabilitation may decrease occurring or increasing care dependency [37]. However, operationalization of the systems and observation time differed, which may explain the lack of significant results in our study.

Our study has some limitations. We used health insurance claims data from 2009–2012, which might be outdated. However, as in Germany there have not been any significant changes of hip fracture treatment since, the results of our analysis should still be valid and relevant. By investigating patients of only one association of health insurance companies, we may introduce a selection bias. There is evidence that insured persons of the AOK may have a lower socio-economic status and are chronically ill more frequently than those of other companies [38, 39]. Therefore, the probability of the increase in care dependency itself may be overestimated. Data on care dependency were only available on a quarterly period basis and it was unclear whether increase in care dependency in the quarterly period of the hip fracture was a consequence of the sustained fracture in all cases. We used the care dependency information in the quarterly period after hip fracture, which may overestimate the risk of occurring or increasing care dependency. A short time horizon was chosen in order to allow for a temporal relation with hip fracture. Studies showed a progressive functional recovery within the first year after hip fracture [40, 41]. We assumed that in case of increase in care dependency after hip fracture, it is likely observed in the first months after hip fracture as the care level determines the reimbursement of both short- and long-term care for the patient. Furthermore, in case of an increased care dependency, the claim is made retroactively as of the date of application and not the date of decision by the MDK, which makes it more likely to be observed shortly after hip fracture. Data were aggregated so that we could not use the patients’ exact residence place but 2-digit postal code regions. Measurement of more precise regional clusters or allowance for geographic characteristics via spatial methods, which would consider adjacent regions, was not possible or useful. Despite the standardized measurement of care dependency in terms of care level, slight differences might be conceivable. On the one hand, the probability of patients applying for a care level, which would initiate the process of assessment, might differ. On the other hand, the realization and practice of the assessment might vary. Although the assessment is conducted by the MDK based on standardized criteria, guidelines and reality might by incongruent which we, however, could neither observe, nor adjust for.

Our study has several strengths. It adds to existing knowledge on care dependency by drawing on a large and rich data set of more than 120,000 patients. We did not exclude patients who died after hip fracture but used mortality as competing risk to account for potential survival bias. We applied generalized linear mixed models to account for autocorrelation of observations within regions. We used claims data, which are less vulnerable to information bias, an issue common for survey data. The AOK has a high national coverage of about one-third of the German population, which makes our results fairly representative and generalizable on patient level. On regional level, our results are specific for Germany and therefore not necessarily generalizable. However, our approach may be adapted to investigate regional variation in other countries. To our knowledge, this is the first study analyzing regional variation of care dependency using the valid classification of German care levels, which clearly discriminates the extent of care dependency. The routinely conducted assessment implies standardized measurement of the degree of care dependency and equal distribution over regions when considering equal patients.

## Conclusion

In this study, we investigated regional variation of occurring or increasing care dependency after hip fractures using the German classification by the long-term care insurance. Several characteristics on patient and regional level associated with care dependency were identified. Knowledge of patient-level characteristics may help to identify possible risk groups, for which special attention may be paid regarding treatment and prevention. Care dependency affects patients’ overall health status and quality of life and it might therefore motivate patients to adopt a preventative lifestyle.

As care dependency is considerably expensive in the long run, driving characteristics might be relevant especially for care providers and payers. We found that patients living in regions with lower hospital density had a slightly higher likelihood for an increase in care dependency after hip fracture. However, the analyses suggested only modest variation on regional level. Differences appear to be attributable primarily to patient rather than regional characteristics.

## References

[1] Kanis JA, Odén A, McCloskey E, Johansson H, Wahl DA, Cooper C. A systematic review of hip fracture incidence and probability of fracture worldwide. Osteoporosis International. 2012;23(9):2239–56. 10.1007/s00198-012-1964-3 22419370 PMC3421108

[2] Cheng S, Levy A, Lefaivre K, Guy P, Kuramoto L, Sobolev B. Geographic trends in incidence of hip fractures: a comprehensive literature review. Osteoporosis International. 2011;22(10):2575–86. 10.1007/s00198-011-1596-z 21484361

[3] Brennan SL, Pasco JA, Urquhart DM, Oldenburg B, Hanna FS, Wluka AE. The association between urban or rural locality and hip fracture in community-based adults: a systematic review. Journal of epidemiology and community health. 2010;64(8):656–65. Epub 2009/08/21. 10.1136/jech.2008.085738 . 19692712

[4] Jacobsen SJ, Goldberg J, Miles TP, Brody JA, Stiers W, Rimm AA. Regional variation in the incidence of hip fracture: US white women aged 65 years and older. Journal of the American Medical Association. 1990;264(4):500–2. 2366282

[5] Alvarez-Nebreda ML, Jimenez AB, Rodriguez P, Serra JA. Epidemiology of hip fracture in the elderly in Spain. Bone. 2008;42(2):278–85. 10.1016/j.bone.2007.10.001 . 18037366

[6] Barbier S, Ecochard R, Schott AM, Colin C, Delmas PD, Jaglal SB, et al Geographical variations in hip fracture risk for women: strong effects hidden in standardised ratios. Osteoporosis International. 2009;20(3):371–7. Epub 2008/07/19. 10.1007/s00198-008-0687-y . 18636217

[7] Defer A, Schober HC, Mohrke W, Abendroth K, Hofbauer LC. Are there still east-to-west differences in the incidence of hip fractures in Germany? Archives of Osteoporosis. 2014;9:195 Epub 2014/10/18. 10.1007/s11657-014-0195-y . 25322672

[8] Wildner M, Clark D. Hip fracture incidence in East and West Germany: reassessement ten years after unification. Osteoporosis International. 2001;12(2):136–9. 10.1007/s001980170146 11303714

[9] Icks A, Arend W, Becker C, Rapp K, Jungbluth P, Haastert B. Incidence of hip fractures in Germany, 1995–2010. Archives of Osteoporosis. 2013;8(1–2):140 10.1007/s11657-013-0140-5 23674147

[10] Bentler SE, Liu L, Obrizan M, Cook EA, Wright KB, Geweke JF, et al The aftermath of hip fracture: discharge placement, functional status change, and mortality. American Journal of Epidemiology. 2009;170(10):1290–9. 10.1093/aje/kwp266 19808632 PMC2781759

[11] Dyer SM, Crotty M, Fairhall N, Magaziner J, Beaupre LA, Cameron ID, et al A critical review of the long-term disability outcomes following hip fracture. BMC geriatrics. 2016;16(1):158 10.1186/s12877-016-0332-0 27590604 PMC5010762

[12] Schulz C, Büchele G, Rehm M, Rothenbacher D, Roigk P, Rapp K, et al Patient Characteristics as Indicator for Care Dependence after Hip Fracture: A Retrospective Cohort Study Using Health Insurance Claims Data From Germany. J Am Med Dir Assoc. 2019;20(4):451–5. Epub November 14, 2018. 10.1016/j.jamda.2018.09.029 30448158

[13] Arbeitsgemeinschaft der Wissenschaftlichen Medizinischen Fachgesellschaften (AWMF). S2e-Leitlinie 012/001: Schenkelhalsfraktur des Erwachsenen 2015 [cited 2019 Jan 10]. Available from: https://www.awmf.org/uploads/tx_szleitlinien/012-001l_S2e_Schenkelhalsfraktur_2015-10_01.pdf.

[14] Bundesministerium für Gesundheit. Zahlen und Fakten zur Pflegeversicherung 2018 [cited 2019 Feb 20]. Available from: https://www.bundesgesundheitsministerium.de/fileadmin/Dateien/3_Downloads/Statistiken/Pflegeversicherung/Zahlen_und_Fakten/Zahlen_und_Fakten.pdf.

[15] Federal Statistical Office Germany. Verzeichnis der Krankenhäuser und Vorsorge- oder Rehabilitationseinrichtungen in Deutschland. Krankenhausverzeichnis. Wiesbaden: Statistische Ämter des Bundes und der Länder; 2011.

[16] Federal Statistical Office Germany. Daten aus dem Gemeindeverzeichnis. Postleitregionen mit regionaler Zugehörigkeit nach Fläche und Bevölkerung. Gebietsstand: 31.12.2011. Wiesbaden: Statistische Ämter des Bundes und der Länder; 2012.

[17] Deutsches Institut für Medizinische Dokumentation und Information. Internationale statistische Klassifikation der Krankheiten und verwandter Gesundheitsprobleme. 10. Revision. German Modification. Köln: Deutschen Institut für Medizinische Dokumentation und Information (DIMDI) im Auftrag des Bundesministeriums für Gesundheit (BMG); 2017.

[18] Becker C, Leistner K, Nikolaus T. Introducing a statutory insurance system for long-term care (Pflegeversicherung) in Germany Michel JP, Rubenstein LZ, Vellas BJ, Albarede JL: Geriatric Programs and departments around the world Serdi-Springer, Paris-New York 1998:55–64.

[19] Häcker J, Hackmann T. Los (T) In Long‐Term Care: Empirical Evidence From German Data 2000–2009. Health Economics. 2012;21(12):1427–43. 10.1002/hec.1805 22081484

[20] Elixhauser A, Steiner C, Harris DR, Coffey RM. Comorbidity measures for use with administrative data. Medical Care. 1998;36(1):8–27. 10.1097/00005650-199801000-00004 WOS:000071181500004. 9431328

[21] van Walraven C, Austin PC, Jennings A, Quan H, Forster AJ. A modification of the Elixhauser comorbidity measures into a point system for hospital death using administrative data. Medical Care. 2009;47(6):626–33. 10.1097/MLR.0b013e31819432e5 19433995

[22] Huber CA, Schneeweiss S, Signorell A, Reich O. Improved prediction of medical expenditures and health care utilization using an updated chronic disease score and claims data. Journal of clinical epidemiology. 2013;66(10):1118–27. 10.1016/j.jclinepi.2013.04.011 23845184

[23] Huber CA, Szucs TD, Rapold R, Reich O. Identifying patients with chronic conditions using pharmacy data in Switzerland: an updated mapping approach to the classification of medications. BMC public health. 2013;13(1):1030 10.1186/1471-2458-13-1030 24172142 PMC3840632

[24] Statistisches Bundesamt Deutschland. Tiefgegliederte Diagnosedaten der Krankenhauspatientinnen und -patienten. Wiesbaden: Statistisches Bundesamt; 2013.

[25] Gardiner JC, Luo Z, Roman LA. Fixed effects, random effects and GEE: what are the differences? Statistics in Medicine. 2009;28(2):221–39. 10.1002/sim.3478 19012297

[26] Neuhaus JM, Kalbfleisch JD. Between-and within-cluster covariate effects in the analysis of clustered data. Biometrics. 1998:638–45. 9629647

[27] Mundlak Y. On the pooling of time series and cross section data. Econometrica: journal of the Econometric Society. 1978:69–85. 10.2307/1913646

[28] Bell A, Fairbrother M, Jones K. Fixed and Random effects models: making an informed choice. Quality & Quantity. 2019;53:1051–74. 10.1007/s11135-018-0802-x.

[29] Ruths S, Baste V, Bakken MS, Engesaeter LB, Lie SA, Haugland S. Municipal resources and patient outcomes through the first year after a hip fracture. BMC health services research. 2017;17(1):144 Epub 2017/02/18. 10.1186/s12913-017-2087-5 28209152 PMC5314693

[30] Fisher ES, Wennberg DE, Stukel TA, Gottlieb DJ, Lucas FL, Pinder EL. The implications of regional variations in Medicare spending. Part 2: health outcomes and satisfaction with care. Annals of Internal Medicine. 2003;138(4):288–98. 10.7326/0003-4819-138-4-200302180-00007 . 12585826

[31] Cary MP Jr., Pan W, Sloane R, Bettger JP, Hoenig H, Merwin EI, et al Self-Care and Mobility Following Postacute Rehabilitation for Older Adults With Hip Fracture: A Multilevel Analysis. Archives of Physical Medicine and Rehabilitation. 2016;97(5):760–71. Epub 2016/02/03. 10.1016/j.apmr.2016.01.012 . 26836951 PMC5823692

[32] Teppala S, Ottenbacher KJ, Eschbach K, Kumar A, Al Snih S, Chan WJ, et al Variation in Functional Status After Hip Fracture: Facility and Regional Influence on Mobility and Self-Care. Journals of Gerontology Series A: Biomedical Sciences and Medical Sciences. 2017;72(10):1376–82. 10.1093/gerona/glw249 28052981 PMC5861914

[33] Hox JJ, Moerbeek M, van de Schoot R. Multilevel analysis: Techniques and applications: Routledge; 2010.

[34] Gilbert R, Todd C, May M, Yardley L, Ben-Shlomo Y. Socio-demographic factors predict the likelihood of not returning home after hospital admission following a fall. Journal of Public Health. 2010;32(1):117–24. Epub 2009/08/12. 10.1093/pubmed/fdp077 . 19666689

[35] Blackburn J, Locher JL, Morrisey MA, Becker DJ, Kilgore ML. The effects of state-level expenditures for home- and community-based services on the risk of becoming a long-stay nursing home resident after hip fracture. Osteoporosis International. 2016;27(3):953–61. Epub 2015/09/25. 10.1007/s00198-015-3327-3 . 26400010

[36] Pitzul KB, Wodchis WP, Carter MW, Kreder HJ, Voth J, Jaglal SB. Post-acute pathways among hip fracture patients: a system-level analysis. BMC health services research. 2016;16:275 Epub 2016/07/20. 10.1186/s12913-016-1524-1 27430219 PMC4950780

[37] Becker C, Rapp K, Rothenbacher D, Schulz C, König H-H, Büchele G. Acute care models for hip fracture treatment vs post-acute rehabilitation services in older adults after hip fracture: A comparative claims data analysis from Germany. Journal of Rehabilitation Medicine. 2019;[Epub ahead of print]. 10.2340/16501977-2630 31748818

[38] Schnee M. Sozioökonomische Strukturen und Morbidität in den gesetzlichen Krankenkassen In: Böcken J, Braun B, Amhof R, editors. Gesundheitsmonitor 2008 Gesundheitsversorgung und Gestaltungsoptionen aus der Perspektive der Bevölkerung. Gütersloh: Verlag Bertelsmann-Stiftung; 2008 p. 88–104.

[39] Hoffmann F, Icks A. Structural differences between health insurance funds and their impact on health services research: results from the Bertelsmann Health-Care Monitor. Gesundheitswesen (Bundesverband der Arzte des Offentlichen Gesundheitsdienstes (Germany)). 2012;74(5):291–7. 10.1055/s-0031-1275711 WOS:000305286100003. 21755492

[40] Magaziner J, Hawkes W, Hebel JR, Zimmerman SI, Fox KM, Dolan M, et al Recovery from hip fracture in eight areas of function. The Journals of Gerontology, Series A: Biological Sciences and Medical Sciences 2000;55(9):M498–M507. 10.1093/gerona/55.9.m498 10995047

[41] Lin P-C, Chang S-Y. Functional recovery among elderly people one year after hip fracture surgery. Journal of Research in Nursing. 2004;12(1):72–82. 10.1097/01.jnr.0000387490.71062.4a 15136965

